# Pandora's Box

**DOI:** 10.1192/bji.2023.29

**Published:** 2023-11

## Hope for ‘chocaholics’?

Research has shown that virtual reality can be a useful method to study consumption behaviour as it can influence thoughts related to food, emotions and behaviour. In a virtual environment, people exposed to food cues have responses such as craving and salivation, and it has been claimed that virtual reality can have more powerful effects on food-related responses than real-life exposure.

In recently published research, the investigators examined the effects of habituation and its possible influence on chocolate consumption. They recruited college students in Singapore, aged 21 to 26 years. In the first study, they examined whether repetitive watching of a 360-degree video of M&M's (chocolate) being consumed could affect the participants’ own consumption behaviour. The participants were assigned to three different conditions: (a) watching a coin being inserted into a laundry machine 33 times (control condition); (b) watching a coin being inserted into a laundry machine 33 times, followed by three repetitions of watching an M&M's chocolate being consumed; and (c) watching three repetitions of a coin inserted into a laundry machine, followed by 30 repetitions of M&M's being consumed. On completion of the tasks, the participants were presented with a bowl of M&M's and were asked to help with a taste test; they were told that they could eat as much as they needed. Their data showed that repeatedly watching a video of M&M's being consumed resulted in the participants consuming fewer M&M's, and they attributed to this to habituation with possible satiation.

In a second study, aiming to produce further confirmation, they included four conditions: three repetitions of watching M&M's being consumed; 30 repetitions of the same; three repetitions of watching a coin being inserted into a laundry machine; and 30 repetitions of the same. This second study confirmed the effects of habituation in reducing consumption of chocolate, with less chocolate consumed after watching 30 repetitions of M&M's being consumed. The control condition of repetitive coin insertion in the laundry machine had no effect on the participants’ chocolate consumption.

So, if you want to cut down on chocolate, repetitive virtual reality exposure may help. Could this also possibly help with food consumption in general?

Li BJ, Lee HM. Exploring the effects of habituation and scent in first-person 360-degrees videos on consumption behaviour. Sci Rep 2023; 13: 835310.1038/s41598-023-35669-5PMC1020573137221377.

## Psychedelics – friend or foe?

Psychedelic drugs, popular with the hippies as well as researchers in the 60s, have seen a rise in interest in recent years with respect to their potential as therapeutic agents. Psychedelics include a variety of drugs with diverse mechanisms of action, namely lysergic acid diethylamide, mescaline, phenylcyclohexylpiperidine, ibogaine, 3,4-methylenedioxymethamphetamine (MDMA), psylocibin and ketamine. What they have in common is that they produce changes to ‘sensory, self, time and space perception that are “so alien to everyday experience that they shed new light on the workings of these everyday mental functions”’. Positive results have been claimed in the treatment of some mental disorders, including addictions, post-traumatic stress disorder and depression. However, psychedelics work through diverse biochemical pathways, targeting different sites on the brain such as serotonin receptor 2A, monoamine transporter, kappa opiate receptor and *N*-methyl-d-aspartate receptor, and until recently no common pathway had been identified to explain their therapeutic properties.

In a recent study, investigators claimed to have discovered a critical period for social reward learning and found that the empathogenic psychedelic MDMA could actually reopen this. Critical periods are specific constrained windows of time in brain development when the nervous system has heightened sensitivity to relevant stimuli, increasing malleability for synaptic, neurocircuit and behavioural modifications. Metaplasticity, a term used to describe the dynamic regulation of the extent to which synaptic plasticity can be induced, is thought to be one of the mechanisms involved in the establishment of critical periods. In a series of experiments, their working model was that psychedelics, acting on different targets in the brain, trigger a downstream signalling response that results in metaplasticity and regulation of the brain extracellular matrix (ECM), which has trophic effects on neuronal cells and affects neurite outgrowth. Both metaplasticity and ECM regulation have been implicated in other critical periods too. The authors suggest that psychedelics could serve as a ‘master key’ to unlock a range of critical periods, enabling modification of various relevant functions. They go as far as suggesting that psychedelics may be beneficial in a wider range of disorders including autism, stroke, blindness and deafness.

Some scientists are encouraged by these results, which offer hope that critical periods are not irreversible and that psychedelic drugs might hold the key to reopening brain plasticity. Other researchers are sceptical of these conclusions, suggesting instead that there is a mechanistic relationship between reopening of critical periods and the altered state of consciousness that is shared by all psychedelics. It is suggested that the drugs could be changing the physical connections between neurons in parts of the brain rather than inducing metaplasticity, which makes the neurons more open to influence by environmental stimuli.

Caution and more research is needed to establish how psychedelics free brain connections and what effects this may have.

Nardou R, Sawyer RE, Song YJ, Wilkinson M, Padovan-Hernandez Y, de Deus JL, et al. Psychedelics reopen the social reward learning critical period. Nature 2023; 618: 790–83731666510.1038/s41586-023-06204-3PMC10284704.

Reardon S. How psychedelic drugs achieve their potent health benefits. Nature 2023; 618: 654–53731660310.1038/d41586-023-01920-2.

## Mind your head

Concussion and its after-effects have attracted increasing interest in relation to sports and in research aimed at gaining a better understanding of the risk factors for sport-related concussion (SRC). The 6^th^ International Conference on Concussion in Sport, held in Amsterdam in October 2022, issued a consensus statement on the subject. The consensus statement was based on systematic reviews carried out over three and a half years. In addition to the consensus statement, the conference produced revised tools, which included the Concussion Recognition Tool-6 and Sport Concussion Assessment Tool-6 (SCAT6, Child SCAT6), as well as a new tool, the Sport Concussion Office Assessment Tool-6.

The consensus statement defined SRC as follows: ‘Sport-related concussion is a traumatic brain injury caused by a direct blow to the head, neck or body resulting in an impulsive force being transmitted to the brain that occurs in sports and exercise related activities. This initiates a neurotransmitter and metabolic cascade, with possible axonal injury, blood flow change and inflammation affecting the brain. Symptoms and signs may present immediately, or evolve over minutes or hours, and commonly resolved within days, but may be prolonged. No abnormality is seen on standard structural neuroimaging studies but in the research setting, abnormalities may be present on functional, blood flow or metabolic imaging studies. Sport-related concussion results in a range of clinical symptoms that may or may not involve loss of consciousness. The clinical symptoms of concussion cannot be explained solely by (but may occur concomitantly with) drug, alcohol, or medication use, other injuries or other comorbidities (such as psychological factors or coexisting medical conditions).’

The latest version of the consensus statement was criticised as ‘their refusal to acknowledge a causal relationship between contact-sports participation and CTE [chronic traumatic encephalopathy] is a danger to the public’. It is pointed out that not many studies have linked repeated sport-related head injuries with CTE. It is also noted that there are serious gaps in the concussion data, which include those in children aged 5 to 12 years, and gender differences. It is important that such studies are carried out, as contact sports – in particular, football – have become increasingly popular among women and young girls.

A recent study examined the association of gender with the incidence and characteristics of concussion. The researchers carried out a prospective longitudinal cohort study, assessing male and female soccer players from all high schools in the Michigan High School Athletic Association (MHSAA) during the academic years 2016–2017 and 2018–2019. SRC data were captured from the MHSAA Head Injury Reporting System, including injury mechanism, immediate management and return to play. Comparisons were made between male and female athletes regarding SRC risk mechanism, short-term management and outcomes, in a total of 43 741 male and 39 637 female soccer athletes who participated in MHSAA soccer during the 3 years of surveillance. Of these participants, 1.8% were reported to have SRC during soccer participation; 37% were boys and 63% were girls. Interestingly, it was noted that boys most often sustained SRC from contact with another player (48.4%), whereas girls’ head injuries were most often from non-player contact (41.9%). Boys were more likely to be removed from play on the day of injury compared with girls, and they typically returned 2 days earlier than the girls. These findings raise a number of questions, i.e. what is the mechanism of the production of concussion in girls compared with boys, and what is the best way to manage injury and risk reduction specifically in female athletes?

Sanderson K. Concussion guidance for sport sidesteps link to brain disease – critics are baffled. Nature 2023; 618: 657–83732225210.1038/d41586-023-01964-4.

Patricios JS, Schneider KJ, Dvorak J, Ahmed OH, Blauwet C, Cantu RC, et al (2023) Consensus statement on concussion in sport: the 6th International Conference on Concussion in Sport – Amsterdam, October 2022. Br J Sports Med 2023; 57: 695–7113731621010.1136/bjsports-2023-106898.

Bretzin AC, Covassin T, Wiebe SJ, Stewart W. Association of sex with adolescent soccer concussion incidence and characteristics. JAMA Netw Open 2021; 4: e21819110.1001/jamanetworkopen.2021.8191PMC808023133904911.

## Beware of climate change and wildfire effects

As wildfires become a frequent phenomenon with climate change, there is increasing concern about their effects on our health. Exposure to wildfires has been linked to cardiovascular, pulmonary and other health problems. The smoke generated contains a large variety of pollutants generated from combustion fuel from sources including our cars and homes. Reports show the presence of neuroinflammation following acute and subchronic exposure to wildfire smoke. This is thought to be related to pulmonary responses to the pollutant, more specifically to pulmonary proteolysis that generates fragmented peptides, which enter the blood circulation and cause damage to the blood–brain barrier, as well as activating microglia and decreasing neuroprotective metabolites.

A study in mice replicating biomass smoke inhalation in the laboratory and using a variety of methods (i.e. flow cytometry, bronchoalveolar lavage and collection of particulate matter characteristics) to explore the timing of onset and resolution of neuroinflammatory and hippocampal metabolomic changes found these to be present for 28 or more days following exposure. These results are in keeping with epidemiological findings of persistent deficits in cognition and attention and prolonged stress and depression after exposure to smoke in wildfires.

Isn't it time to take the effects of climate change and pollution more seriously?

Scieszka D, Jin Y, Noor S, Barr E, Garcia M, Begay J, et al. Biomass smoke inhalation promotes neuroinflammatory and metabolomic temporal changes in the hippocampus of female mice. J Neuroinflammation 2023; 20: 19210.1186/s12974-023-02874-yPMC1046413237608305.

